# The Impact of Following Instagram Influencers on Women’s Body Dissatisfaction and Eating Disorder Symptoms

**DOI:** 10.3390/nu16162730

**Published:** 2024-08-16

**Authors:** Sara Bocci Benucci, Giulia Fioravanti, Valeria Silvestro, Maria Chiara Spinelli, Giulietta Brogioni, Alessia Casalini, Lara Allegrini, Arianna Ida Altomare, Giovanni Castellini, Valdo Ricca, Francesco Rotella

**Affiliations:** 1 Department of Experimental and Clinical Medicine, University of Florence, Largo Brambilla, 3, 50134 Florence, Italy; sara.boccibenucci@unifi.it; 2 Psychology Unit, Department of Health Sciences, University of Florence, Via di San Salvi 12, 50135 Florence, Italy; giulia.fioravanti@unifi.it; 3 Psychiatry Unit, Department of Health Sciences, University of Florence, Largo Brambilla 3, 50134 Florence, Italy; dietista.valeriasilvestro@gmail.com (V.S.); maria.spinelli@edu.unifi.it (M.C.S.); giulietta.brogioni@unifi.it (G.B.); alessia.casalini1@edu.unifi.it (A.C.); lara.allegrini@unifi.it (L.A.); ariannaida.altomare@unifi.it (A.I.A.); giovanni.castellini@unifi.it (G.C.); valdo.ricca@unifi.it (V.R.)

**Keywords:** body dissatisfaction, dieting, eating disorders, fitness, influencers, Instagram, social media

## Abstract

According to the Tripartite Influence Model, social media is one of the primary sources influencing women’s body dissatisfaction. However, the role of social media influencers as a potential driver for impacting users’ body image evaluation when disseminating content on social networks has been little investigated. The present research aims to explore the relationship between following three Instagram influencers (i.e., nutrition, fitness, and entertainment) and eating disorder (ED) symptoms and body dissatisfaction among a group of female users. A sample of 5060 women (mean age = 35.33 ± 9.33) were recruited with the collaboration of three Italian influencers, and self-reported questionnaires were administered. Women who followed the nutritional influencer account reported significantly higher scores on ED symptoms and body dissatisfaction than women who followed the fitness and entertainment influencer accounts (η^2^ = 0.05 and η^2^ = 0.02, respectively). Overall, following nutrition and fitness accounts (compared to entertainment accounts) and spending more time daily on social networks positively predicts ED symptoms (β = 0.28, *p* < 0.001, β = 0.10, *p* < 0.001, β = 0.11, *p* < 0.001, respectively) and body dissatisfaction (β = 0.07, *p* < 0.001, β = 0.04, *p* < 0.001, β = 0.07, *p* < 0.001, respectively). Moreover, following nutritional influencers compared to fitness influencers positively predicts ED symptoms (β = 0.17, *p* < 0.001) but not body dissatisfaction. The current results suggest that being exposed to dieting and weight loss topics on social media might be particularly harmful for individuals with specific vulnerabilities. Practical implications will be discussed.

## 1. Introduction

According to the Tripartite Influence Model (also named the sociocultural model of body dissatisfaction) [1], the primary sources influencing body dissatisfaction are peers, parents, and media, the latter being the most impactful. In the realm of new media, Social Network Sites (SNSs) are the most used and differ from traditional media by the possibility of creating one’s user profile, the higher interactivity, the possibility to share content with other users, and maintaining existing relationships as well as creating new ones [2]. Even though the use of SNSs can be beneficial when, for example, it allows users to experience higher social connectedness [3], a growing body of literature has found a negative impact of using and being exposed to SNSs on body image. Indeed, like traditional media, SNSs are often appearance-focused since users post photos in which they look good and attractive, enhanced by the application of filters or digital editing tools and poses [4]. As a result, many of the images presented on SNSs are idealized and unrealistically attractive, thus inducing body dissatisfaction. In support of this, a consistent body of research has shown that SNS use is associated with body image concerns and disordered eating [5,6,7]. Internalization of beauty ideals, appearance-based comparison, and self-objectification were found to explain the detrimental effect of SNS use on body image [5,6].

The new professional figure of social media influencers (SMIs) could strongly impact users’ body image evaluation when disseminating content on social networks. Influencers can be defined as vocational content creators who amass large followings on social media and monetize their activity over time [8,9]. Influencers often choose a particular area of communication (e.g., fitness, travel, makeup, food, etc.), and on SNSs, they are considered celebrities [9]. Moreover, SMIs and their followers are engaged in a mediated relationship (SMIs frequently interact with social media users, and then followers have the impression they know them well), and there is evidence that perceived closeness of this relationship influences the effectiveness of SMIs’ messages [10,11].

According to the sociocultural model [1], celebrities can contribute to the depiction of unrealistic beauty standards through SNSs, and their followers could drive to achieve the body they are exposed to online. Research found that being exposed to attractive celebrity pictures harms mood and women’s body image [12]; specifically, women reported higher body dissatisfaction, lower body appreciation [13], and lower weight satisfaction [14]. Moreover, exposure to both thinspiration (i.e., ideal images representing thin bodies) and fitspiration (i.e., images of thin and toned bodies, posing in fitness clothing or engaging) content on Instagram is also particularly harmful to women’s body image [5]. A recent scoping review investigating the impact of influencers on adolescents’ health found that due to the established trustworthy relationship with their audience, the major risk is when influencers promote unrealistic body image, unhealthy diets, substance use, inaccurate diagnosis, and treatment advice [8].

In the last decade, health professionals have recognized SNSs’ potential to share health knowledge and promote healthy behaviors. For instance, the category of nutritional influencers is represented by highly followed nutritional professionals (nutritionists, dietitians) who share scientific information about nutrition and related aspects (e.g., dieting and exercising) on their accounts. Fitness influencers usually have a qualification related to physical activity or physical health (e.g., personal trainer, physiotherapy); share exercise videos, example workouts, and targeted exercise motivation (e.g., improving strength, improving appearance, reaching specific goals); and encourage healthy eating [15]. Although some studies have proven that fitness and nutritional influencers positively affect people’s exercise intentions or behaviors and eating habits [16], engaging with fitspiration material and following accounts of dietitians/nutritionists was positively associated with disordered eating and body dissatisfaction [17,18,19].

Moreover, previous research has shown that many health influencers who share health and nutrition advice on social media are poorly qualified and promote unhealthy nutrition messages [20]. Similarly, a recent study [15] found that nearly two-thirds of the 100 most popular fitness influencer accounts promoted unhealthy or unrealistic body shapes. This calls for further research into the influence of nutritional and fitness influencers on disordered eating symptomatology and body image concerns to understand better whether the eating-related content and exercise education they provide may harm viewers’ eating behaviors and body image.

Therefore, the aim of the present research is to explore the relationship between following three social media influencers (i.e., nutrition, fitness, and entertainment influencers) and eating disorder (ED) symptoms and body dissatisfaction among a group of female Instagram users.

## 2. Materials and Methods

### 2.1. Participants and Procedure

A convenience sampling approach was used to recruit a sample of 5060 (mean age = 35.33 ± 9.33; age range 18–65) female Instagram users. The sample was mainly composed of employees (60.7%), followed by self-employees/freelancers (18.2%), students (12.5%), and unoccupied people (8.1%). The remaining 0.7% were retired.

Concerning educational background, the majority had a master’s degree (36.3%), followed by a high school diploma (35.4%), bachelor’s degrees (23.1%), and a middle school diploma (3.4%), and the remaining had a PhD or a higher qualification (1.9%). Additionally, the mean BMI of the total sample was 23.77 ± 4.86 Kg/m^2^.

Participants were recruited through Instagram with the free collaboration of three Italian influencers: @sara.postura.da.paura (Sara Compagni, fitness influencer), @bilanciamo (Giulia Biondi, nutritionist influencer), and @ireneccloset (Irene Colzi, entertainment influencer). Some criteria were followed to select the influencers. In general, to be selected for the first screening, the influencers needed to satisfy the following criteria: (a) being female, (b) having at least 10 k followers, (c) creating content in Italian and addressing an Italian-speaking audience. In addition, specific criteria were set for each category of influencer. For the fitness influencer, the creator had to meet the following criteria:

- Appear in Instagram searches if at least one of the following keywords was entered: “fit”, “healthy”, “fitness”, “workout”, “fitspo”.
- Daily publication of content relating to physical activity.
- Must not be nutrition professionals (dieticians, nutritional biologists, nutritional doctors) or entertainment influencers.

For the nutrition professional influencer, the creator had to meet the following criteria:

- Appear in Instagram searches if at least one of the following keywords was entered: “diet”, “dietician”, “nutritionist”, “nutrition”, “healthy eating”.
- Daily publication of content relating to nutrition.
- Must be licensed nutrition professionals.

For the entertainment influencer, the creator had to meet the following criteria:

- Daily publication of content relating to three or more of the following topics: comedy/entertainment, daily life events, TV series, music, books, entertainment.
- Must not be nutrition professionals and/or fitness influencers.

Each influencer was instructed to post three Instagram stories on a predetermined date and time. The first story included a call-to-action button, the second briefly explained the study using a pre-established common text, and the third contained a link to the informed consent form and the study questionnaire. Participants were informed that participation was voluntary and anonymous, and that confidentiality was guaranteed. The questionnaire comprised demographic information, anthropometric information (i.e., weight and height), if they had an eating disorder diagnosis during their lifetime, questions concerning Instagram use and two psychometrically sound scales assessing symptoms and concerns typical of EDs and body dissatisfaction.

All the participants consented to participate in this study, and the questionnaire was administered through Google Forms. Compilation took approximately 10 min. No remunerative rewards were given. The study procedures were carried out in accordance with the Declaration of Helsinki and approved by the Committee for the Ethics of Research of the University of Florence.

### 2.2. Measures

#### 2.2.1. Eating Attitudes Test–26

The Italian version [21] of the Eating Attitudes Test–26 (EAT-26) [22] is a 26-item self-report questionnaire which assesses symptoms and concerns typical of EDs. The scale is presented along three dimensions: dieting (referring to the restricted intake of high-caloric food and concerns with body image/shape), bulimia and food preoccupation (thoughts regarding food, binging and self-induced vomiting), and oral control (concerning the ability to regulate food intake and managing perceived pressure from others to gain weight). A sample item for each scale is “I am aware of the calorie content of foods that I eat.” (dieting), “I vomit after I have eaten” (bulimia and food preoccupation), “I feel that others would prefer if I ate more” (oral control). Items are presented on a 6-point Likert scale ranging from 1 (never) to 6 (always), with higher scores reflecting higher concerns and more severe symptomatology. Moreover, the scale presents a cut-off score (i.e., a score of 20 or higher) that identifies individuals at risk for EDs. In the current sample, Cronbach’s’ alphas and McDonald’s omegas were α = 0.85, ω = 0.87 (dieting), α = 0.78, ω = 0.87 (bulimia and food preoccupation), α = 0.66, ω = 0.64 (oral control), and α = 0.89, ω = 0.91 (total score), suggesting good internal consistency.

#### 2.2.2. Stunkard Figure Rating Scale

The Italian version [23] of the Stunkard Figure Rating Scale [24] is a 2-item self-report scale that assesses one’s current body shape and size, as well as the ideal body shape and size, and the discrepancy between the second and first responses is a measure of body dissatisfaction. The scale presents nine silhouettes drawn for males and females varying in body dimension and shape (from the thinnest to the largest). Each participant is asked to indicate which silhouette they perceive as the most accurately depicting their body size and which silhouette they perceive as the most accurately depicting their ideal body size. In the current sample, Cronbach’s’ alpha was 0.68.

#### 2.2.3. Ad Hoc Questions Assessing Instagram Use

Even though it was possible to identify from which influencer participants found the questionnaire through a filter question, some ad hoc questions were asked to investigate participants’ Instagram use. Specifically, they were asked to indicate if they also followed the two types of influencers other than the one they found the link in stories from, which type of influencers they followed the most, their time spent on Instagram per day, the time spent on fit/nutrition/entertainment content per day, and the frequency of posting photos of themselves.

### 2.3. Statistical Analysis

Participants were divided into three sub-samples according to the type of influencer from whom they found the questionnaire (e.g., fitness influencer, nutrition influencer, entertainment influencer). After performing descriptive statistics, a series of chi-squares were performed to explore differences in demographics and Instagram use in the three sub-samples. Subsequently, a series of one-way Analyses of Variance (ANOVAs) with the Bonferroni post hoc test was performed to explore differences between the fitness/nutrition/entertainment influencer samples in age, BMI, ED symptoms, and body dissatisfaction. Subsequently, correlations between the study variables and a series of multiple regression analyses in the total sample were performed to investigate if age, BMI, time spent on Instagram daily, and the type of influencers followed could predict (i) EAT-26 total score, (ii) dieting, (iii) bulimia and food preoccupation, (iv) oral control, and (v) body dissatisfaction.

All the analyses were performed using IBM Statistical Package for the Social Sciences (SPSS), version 28.0 (IBM Corp., Armonk, NY, USA).

## 3. Results

### 3.1. Chi-Square Differences between the Three Sub-Samples

Table 1 shows differences between the three sub-samples recruited from the different types of influencers on demographic characteristics, Instagram use, and frequency of having received an ED diagnosis and being at risk for EDs (i.e., EAT-26 score ≥ 20).

Education levels are lower in the fitness influencer sub-sample than in the other two. Women who follow entertainment influencers spend more time on Instagram daily than women in the nutrition and fitness influencer samples.

Among nutritional influencer followers, more women have received an ED diagnosis and are more at risk for an ED compared to women who follow the other two types of influencers.

### 3.2. One-Way ANOVAs Differences between the Three Sub-Samples

Significant differences emerged between the three sub-samples (see Table 2). Specifically, the nutrition influencer sub-sample reported higher scores for all the variables assessed compared to the fitness and entertainment influencer sub-samples, while the fitness influencer sub-sample reported significantly higher scores than the entertainment influencer sub-sample for age, dieting, bulimia/food preoccupation, and total EAT-26 score.

### 3.3. Correlations and Regression Analyses in the Total Sample

Table 3 displays Pearson’s correlations among the study variables, showing all significant correlations except for age with bulimia/food preoccupation and with EAT-26 total score and for BMI with time spent on Instagram per day.

Regression analyses were conducted to investigate if age, BMI, time spent on Instagram daily, and the type of influencers from whom participants found the questionnaire (e.g., fitness influencer, nutrition influencer, entertainment influencer) could predict (i) ED total symptoms, (ii) dieting, (iii) bulimia and food preoccupation, (iv) oral control, and (v) body dissatisfaction. Results are reported in Table 4 and Table 5. Overall, being younger, having a higher BMI, following nutrition and fitness accounts (compared to entertainment accounts), and spending more time a day on SNSs positively predicts ED symptoms. Following nutritional influencers emerged as the strongest predictor. Regarding body dissatisfaction, being older, having a higher BMI, following nutritional and fitness accounts (compared to entertainment accounts), and spending more time a day on SNSs emerged as significant predictors, with BMI being the strongest. Moreover, following nutritional influencers compared to fitness influencers positively predicts ED symptoms (but not body dissatisfaction).

## 4. Discussion

The current study explores the impact of following nutritional, fitness, and entertainment accounts on female Instagram users’ body dissatisfaction and eating disorder symptoms. Three influencers recruited the sample, which was divided into three sub-samples according to which of the three Instagram accounts they found the link for completing the questionnaire through.

Significant differences emerged between the three sub-samples. Specifically, participants in the nutritional sub-sample had a higher mean BMI that fit into the range of overweight (i.e., a BMI between 24.9 and 29.9) compared to that of the fitness and entertainment samples, which was within the normal weight range (i.e., a BMI between 18.5 and 24.8). In addition, the nutritional sub-sample spent significantly more time on SNSs than the other two groups. Regarding screening for EDs, participants in the nutritional influencer sub-sample more frequently declared they had received a diagnosis of ED in their lifetime or more frequently scored 20 or above in the EAT-26, which identifies individuals at risk for an eating disorder. Lastly, the nutritional sub-sample reported significantly higher scores than the other two sub-samples in dieting, bulimia and food preoccupation, oral control, ED symptoms and concerns (i.e., EAT26 total score), and body dissatisfaction. These results confirm previous evidence about the negative impact of following accounts of dietitians/nutritionists on women’s body image and eating behaviors [17]. On the other hand, these results could shed light on the more frequent characteristics of female users who are more interested in following nutritional content. Indeed, it is also possible to hypothesize that individuals with an existing vulnerability to body dissatisfaction and ED symptoms might be more interested in content that promotes aspects related to weight loss. Reviews on how nutrition is communicated on SNSs by influencers report that the most frequent theme regards promoting a dietary change, especially through restrictive diets [25]. In line with what was previously recommended by Rounsefell et al. [26], it seems to be extremely important that nutritional influencers pay attention to not defeat the original intent of health messages to follow social marketing strategies because of the risk of unintentionally perpetuating body dissatisfaction and disordered eating in both at-risk and healthy individuals.

The fitness influencer sub-sample scored significantly higher than the entertainment influencer sub-sample in dieting, bulimia and food preoccupation, and ED symptom total score. Previous research investigated the impact of being exposed to fitspirational content on SNSs on women’s body image, showing that viewing fitspirational images negatively impacts women’s body image [27] compared to being exposed to neutral images. In addition, previous research analyzing fitspiration contents showed that for some aspects, including dieting, these contents did not significantly differ from the thinspirational ones [25]. Research investigating the link between ED symptom severity and exposure to thinspiration and fitspiration content found that thinspiration content was strongly correlated to ED symptom severity (r = 0.40, *p* < 0.001) and that fitspiration content was positively associated with ED symptom severity (r = 0.29, *p* < 0.001); additionally, both were linked to more frequent use of image-centered SNSs, and exposure to fitspiration was more common than exposure to thinspiration content [28]. Our results seem to be in line with these observations, suggesting that higher scores on dieting, bulimia and food preoccupation, and ED symptoms and concerns found in individuals following fitness influencers’ accounts compared to the entertainment influencer followers could be in part related to the exposure to the type of content and messages promoted in fitspiration accounts. Indeed, fitspiration content on SNSs often disseminates unrealistic and ostensible bodies in which pictures of “before-and-after” exercising do not seem to promote the benefits of exercising on health [29], but several problematic behaviors such as extreme exercising, counting calories, fasting, and consuming dietary supplements without medical supervision [29,30,31].

In addition, regression analyses showed that the strongest predictor of the total score of the EAT-26 and the related subscales (i.e., dieting, bulimia and food preoccupation, oral control) was following nutritional and fit influencers (compared to entertainment influencers), followed by higher BMI, time spent on SNSs, and being younger. Notably, when some well-known risk factors for EDs (e.g., younger age and higher BMI) are included in the model, the impact of social networks maintains its significance, thus suggesting its prominent role. This is in line with previous research supporting the impact of media [32] and social media [33,34] in the onset and maintenance of eating disorder symptoms. Moreover, following nutritional influencers also emerged as a significant predictor of ED symptoms when compared to following fitness influencers. This result could be partially explained by the fact that nutritional influencers’ followers are more exposed to dieting and weight loss topics, which might be particularly harmful to individuals with specific vulnerabilities (e.g., internalization of thin beauty ideals, appearance-based comparison, and self-objectification) [5,6].

Concerning predictors of body dissatisfaction, the same results discussed above emerged, except that having a higher BMI was the strongest predictor and being older rather than younger predicted higher body dissatisfaction. This is consistent with previous research in which higher BMI and older age predict greater body dissatisfaction and weight and shape concern [35,36]. In this case, following nutritional influencers did not predict body dissatisfaction compared to following fitness influencers, suggesting that the (negative) impact on body image is the same for both types of influencers’ followers. This could be explained by the fact that regardless of the topic communicated, influencers propagate an idealized (and unattainable) body shape [30].

The majority of previous research investigated the impact of SNS use on normal-weight adolescents and young adults’ body image, neglecting diverse samples of older women, and women with underweight or overweight body sizes who, in any case, are among the users of Instagram [6,37]; the current results shed light on the importance of considering the impact of following certain types of influencers’ accounts on SNSs on adult women’s body image with different body sizes. Moreover, the current study builds upon previous research by exploring the impact of the new professional figures of nutritional and fitness influencers when disseminating specific content on social networks on viewers’ eating behaviors and body image. In particular, this research takes a step forward in exploring the impact of following nutritional influencers’ accounts (compared to the better previously explored fitness influencers) on Instagram on women’s body dissatisfaction and ED symptoms.

Some limitations of this study should be addressed.

First, the cross-sectional design does not allow us to make cause-and-effect inferences. Future research could implement longitudinal and experimental designs to explore how exposure to nutritional and fitness content through social media influencers could impact women’s body image.

Second, the convenience sampling approach could limit the generalizability of the results. In addition, involving influencers in the recruitment process, even though it helped to recruit well-targeted individuals, could have inserted some response bias in the sample. Future research should include more than one influencer for each category explored and use random sampling approaches.

Furthermore, although we controlled for time spent on Instagram in the regression analyses, we did not control for the type of Instagram use. This is a relevant shortcoming, as some women might be exposed to fitspiration content in the nutritional influencer sub-sample and vice versa. Indeed, different content (i.e., nutrition, fitness, entertainment) always exists in the daily use of Instagram and may have a constant effect during everyday social media use. In this context, women may experience their psychological well-being as a balance between negative effects related to exposure to body-related content and (positive) effects related to exposure to appearance-neutral content. In this vein, it could be methodologically useful to differentiate between unique types of social media influencer exposure (i.e., nutritional, fitness, and entertainment) to best capture the influence of nutrition/fitness content on body image and disordered eating.

Despite these limitations, these results could have several practical implications. First, the impact of viewing nutritional and fitspiration content on women’s body dissatisfaction and ED symptoms and concerns has also been confirmed in women aged 25–35 years old. Previous research has focused on adolescents and young adults [8,30,38]; these results confirm that viewing nutritional and fitspiration content on SNSs could also be detrimental for adult women, suggesting considering the influence of following social media influencer accounts in the assessment of adult women with ED symptoms and/or body image disturbance. In addition, results that emerged on the nutritional sample shed light on the importance of nutritional influencers paying attention to the messages they spread with their content because there is a risk of triggering body dissatisfaction and ED symptoms and concerns in vulnerable individuals. Since the spread of health professional influencers is a recent phenomenon, the general need for more regulation of online communication for health-related topics emerges so that those health professionals who aspire to work as influencers do not have to pursue only marketing strategies but have health promotion as their main goal. A practice paper from the Academy of Nutrition and Dietetics [39] provides guidance on best practices for dietetics practitioners on social media. Among them, it suggests following the 80/20 rule when determining what to share on social media (i.e., 80% of social media content should benefit the audience and 20% should be self-promotional) and adding value by providing updates on new nutrition studies and sharing content created by food and nutrition peers and the Academy.

A recent review [26] suggests that social media health messages should avoid focusing on weight or body aesthetics and instead promote body functionality (i.e., everything the body can do or is capable of doing). Future research could investigate the impact of following accounts promoting body functionality messages on women’s body image and study which type of communication and messages delivered by influencers could promote functionality appreciation and positive body image.

## 5. Conclusions

The current results suggest that following nutritional and fitness influencers on Instagram negatively impacts women’s body image evaluation and eating behaviors.

Furthermore, following nutritional influencers emerged as a significant predictor of ED symptoms when compared to following fitness influencers. This result could be partially explained by the fact that being exposed to dieting and weight loss topics on social media might be particularly harmful to individuals with specific vulnerabilities (e.g., internalization of thin beauty ideals, appearance-based comparison, and self-objectification). It is extremely important that nutritional influencers pay attention to the messages they spread when disseminating specific content on social networks because there is a risk of triggering body dissatisfaction and ED symptoms and concerns in vulnerable individuals.

## Figures and Tables

**Table 1:** Descriptive statistics and chi-square differences between the three samples.

	Fitness Influencer Sample (N = 1439)	Nutrition Influencer Sample (N = 1672)	Entertainment Influencer Sample (N = 1949)	χ2 _(df)_	*p*
***Demographics***	n	n	n		
**Education**				2206.014 (10)	<0.001
Middle school diploma	628	120	24		
High school diploma	403	744	637		
Bachelor’s degree	371	288	500		
Master’s degree	0	487	760		
PhD or higher qualification	37	33	28		
**Occupation**				182.806 (10)	<0.001
Worker	923	937	1211		
Self-employed/freelancer	269	340	307		
Student	176	142	312		
Unoccupied	64	231	114		
Retired	7	22	5		
***Instagram use***	n	n	n		
**Time spent on Instagram a day**				178.847 (6)	<0.001
Less than one hour	192	467	243		
1–2 h	878	888	1214		
3–4 h	324	272	434		
More than 4 h	45	45	58		
**Time spent a day on fitness content**				268.991 (8)	<0.001
I do not follow fit content	180	489	701		
Less than one hour	1129	1017	1154		
1–2 h	128	154	88		
3–4 h	2	11	6		
More than 4 h	0	1	0		
**Time spent a day on nutritional contents**				492.655 (8)	<0.001
I do not follow nutritional content	178	56	514		
Less than one hour	1120	1288	1311		
1–2 h	137	308	120		
3–4 h	4	19	4		
More than 4 h	0	1	0		
**Time spent a day on entertainment content**				442.317 (8)	<0.001
I do not follow entertainment content	65	223	24		
Less than one hour	748	988	849		
1–2 h	571	414	943		
3–4 h	50	39	129		
More than 4 h	5	8	4		
**Frequency of posting photos of oneself**				91.037 (6)	<0.001
Never	809	998	910		
Less than one time a week	586	581	956		
1–4 times a week	35	85	69		
Every day	9	8	14		
**Have you ever received an ED diagnosis?**				48.252 (2)	<0.001
Yes	162	289	179		
No	1291	1473	1787		
**At risk for Eds (EAT-26 score ≥ 20)**				145.826 (2)	<0.001
Yes	214	425	191		
No	1239	1337	1775		

**Table 2:** ANOVAs between the three samples on age, BMI, and body image-related variables.

	Fitness Influencer (N = 1439)	Nutritional Influencer (N = 1672)	Entertainment Influencer (N = 1949)	F(df = 2)	*p*	η^2^	Bonferroni Post Hoc Test
	M ± SD	M ± SD	M ± SD				
Age (years)	34.72 ± 8.92	39.88 ± 10.07	31.88 ± 7.08	386.618	<0.001	0.13	“Nutritional” > “Fit” > “Entertainment”
BMI (kg/m^2^)	23.09 ± 3.90	24.94 ± 5.72	23.27 ± 4.50	74.731	<0.001	0.03	“Nutritional” > “Fit” & “Entertainment”
Dieting	6.27 ± 6.38	8.44 ± 7.30	4.82 ± 5.60	143.589	<0.001	0.05	“Nutritional” > “Fit” > “Entertainment”
Bulimia and Food preoccupation	1.93 ± 2.97	2.81 ± 3.55	1.36 ± 2.61	102.467	<0.001	0.04	“Nutritional” > “Fit” > “Entertainment”
Oral control	0.86 ± 1.65	1.22 ± 2.17	0.89 ± 1.80	18.713	<0.001	0.007	“Nutritional” > “Fit” & “Entertainment”
ED symptoms and concerns (total EAT26)	9.16 ± 9.79	12.63 ± 11.64	7.17 ± 8.62	134.515	<0.001	0.05	“Nutritional” > “Fit” > “Entertainment”
Body dissatisfaction	1.46 ± 1.29	1.84 ± 1.38	1.36 ± 1.29	63.533	<0.001	0.02	“Nutritional” > “Fit” & “Entertainment”

**Table 3:** Correlations among the study variables in the total sample (N = 5060).

	M ± SD	1.	2.	3.	4.	5.	6.	7.	8.
1. Age (years)	35.33 ± 9.33	-	0.23 **	0.03 *	−0.002	−0.09 **	0.002	0.18 **	−0.23 **
2. BMI (kg/m^2^)	23.77 ± 4.86		-	0.16 **	0.22 **	−0.13 **	0.14 **	0.58 **	0.01
3. Dieting	6.43 ± 6.60			-	76 **	0.42 **	0.96 **	0.39 **	0.08 **
4. Bulimia and food preoccupation	2 ± 3.11				-	0.35 **	0.86 **	0.38 **	0.12 **
5. Oral control	0.99 ± 1.90					-	0.58 **	−0.04 **	0.06 **
6. ED symptoms and concerns (EAT26 total score)	9.54 ± 10.29						-	0.35 **	0.10 **
7. Body dissatisfaction	1.55 ± 1.34							-	0.06 **
8. Time spent on Instagram daily (hours)	2.08 ± 0.70								-

* *p* < 0.05; ** *p* < 0.001.

**Table 4:** Regression analyses with “following entertainment influencers” as the reference category.

Criterion Variable	ED Symptoms and Concerns		Dieting		Bulimia and Food Preoccupation		Oral Control		Body Dissatisfaction	
	β	t	β	t	β	t	β	t	β	t
Age	−0.09 **	−6.092	−0.06 **	−4.352	−0.10 **	−6.744	−0.09 **	−6.207	0.05 **	4.205
BMI	0.12 **	8.473	0.14 **	10.016	0.21 **	15.543	−0.14 **	−9.657	0.56 **	47.325
Time spent on Instagram daily	0.11 **	8.264	0.10 **	7.067	0.13 **	9.431	0.06 **	4.303	0.07 **	6.245
Following nutrition influencers (0 = entertainment)	0.28 **	17.545	0.28 **	17.064	0.24 **	15.276	0.15 **	9.265	0.07 **	4.897
Following fitness influencers (0 = entertainment)	0.10 **	6.278	0.11 **	7.314	0.10 **	6.790	0.005	0.348	0.04 *	2.798
**% of variance explained**	8%		8%		11%		4%		34%	

* *p* < 0.05; ** *p* < 0.001.

**Table 5:** Regression analyses with “following fitness influencers” as the reference category.

Criterion Variable	ED Symptoms and Concerns		Dieting		Bulimia and Food Preoccupation		Oral Control		Body Dissatisfaction	
	β	t	β	t	β	t	β	t	β	t
Age	−0.09 **	−5.918	−0.06 **	−4.081	−0.09 **	−6.604	−0.10 **	−6.419	0.06 **	4.889
BMI	0.11 **	8.359	0.14 **	10.020	0.21 **	15.456	−0.14 **	−9.958	0.55 **	47.279
Time spent on Instagram daily	0.11 **	7.973	0.09 **	6.657	0.13 **	9.293	0.06 **	4.055	0.07 **	6.354
Following nutrition influencers (0 = fitness)	0.17 **	10.136	0.15 **	9.065	0.13 **	7.833	0.15 **	8.692	0.01	0..970
Following entertainment influencers (0 = fitness)	−0.11 **	−6.708	−0.12 **	−7.297	−0.11 **	−6.784	−0.006	−0.351	−0.04 *	−2.586
**% of variance explained**	8%		8%		10%		4%		34%	

* *p* < 0.05; ** *p* < 0.001.

## Data Availability

The original contributions presented in the study are included in the article, further inquiries can be directed to the corresponding author.
